# Multiple Promoters and Alternative Splicing: *Hoxa5* Transcriptional Complexity in the Mouse Embryo

**DOI:** 10.1371/journal.pone.0010600

**Published:** 2010-05-12

**Authors:** Yan Coulombe, Margot Lemieux, Julie Moreau, Josée Aubin, Milan Joksimovic, Félix-Antoine Bérubé-Simard, Sébastien Tabariès, Olivier Boucherat, François Guillou, Christian Larochelle, Christopher K. Tuggle, Lucie Jeannotte

**Affiliations:** 1 Centre de recherche en cancérologie de l'Université Laval, Centre Hospitalier Universitaire de Québec, L'Hôtel-Dieu de Québec, Québec, Québec, Canada; 2 Department of Animal Science, Iowa State University, Ames, Iowa, United States of America; National University of Singapore, Singapore

## Abstract

**Background:**

The genomic organization of *Hox* clusters is fundamental for the precise spatio-temporal regulation and the function of each *Hox* gene, and hence for correct embryo patterning. Multiple overlapping transcriptional units exist at the *Hoxa5* locus reflecting the complexity of *Hox* clustering: a major form of 1.8 kb corresponding to the two characterized exons of the gene and polyadenylated RNA species of 5.0, 9.5 and 11.0 kb. This transcriptional intricacy raises the question of the involvement of the larger transcripts in *Hox* function and regulation.

**Methodology/Principal Findings:**

We have undertaken the molecular characterization of the *Hoxa5* larger transcripts. They initiate from two highly conserved distal promoters, one corresponding to the putative *Hoxa6* promoter, and a second located nearby *Hoxa7*. Alternative splicing is also involved in the generation of the different transcripts. No functional polyadenylation sequence was found at the *Hoxa6* locus and all larger transcripts use the polyadenylation site of the *Hoxa5* gene. Some larger transcripts are potential *Hoxa6*/*Hoxa5* bicistronic units. However, even though all transcripts could produce the genuine 270 a.a. HOXA5 protein, only the 1.8 kb form is translated into the protein, indicative of its essential role in *Hoxa5* gene function. The *Hoxa6* mutation disrupts the larger transcripts without major phenotypic impact on axial specification in their expression domain. However, *Hoxa5*-like skeletal anomalies are observed in *Hoxa6* mutants and these defects can be explained by the loss of expression of the 1.8 kb transcript. Our data raise the possibility that the larger transcripts may be involved in *Hoxa5* gene regulation.

**Significance:**

Our observation that the *Hoxa5* larger transcripts possess a developmentally-regulated expression combined to the increasing sum of data on the role of long noncoding RNAs in transcriptional regulation suggest that the *Hoxa5* larger transcripts may participate in the control of *Hox* gene expression.

## Introduction

*Hox* genes play a crucial role in specifying regional identity along the body axes and in regulating morphogenesis during animal development. Inappropriate expression and mutation of *Hox* genes can disrupt normal programs of growth and differentiation leading to malformations, tumor formation, and even death [1]. In mammals, 39 *Hox* genes are distributed over four clusters sharing a similar organization that reflects the relationship existing between the relative position of each *Hox* gene along the cluster, its expression domain in the embryo and its temporal onset. *Hox* genes have RNA expression domains extending from the caudal end of the embryo to a defined anterior limit. The resulting spatio-temporal profile of *Hox* gene expression during embryogenesis correlates with the arrangement of the clusters: the 3′ most genes being expressed earlier and in more anterior domains than the 5′ located ones [2]. Consequently, the clustered organization appears fundamental for the precise spatio-temporal regulation and the function of each *Hox* gene and hence for the correct patterning of the embryo.

How *Hox* gene expression is modulated along the developing axes still remains elusive. Our initial knowledge of the regulatory mechanisms governing *Hox* gene expression comes mostly from transgenic mice studies, which have shown that *Hox* dynamic expression patterns result from positional information transducing via transcription factors that interact with a combination of positive and negative *cis*-acting sequences to differentially control *Hox* gene expression in a spatio-temporal and tissue-specific fashion. However in most cases, only limited subsets of the proper spatial and temporal expression patterns are reconstituted by the transgenes. A likely explanation is the presence of complex and overlapping transcriptional units in *Hox* genes that implies dispersed regulatory regions in the clusters [3]–[5]. There is also evidence for the integrated regulation of neighboring *Hox* genes through the sharing, the competition and/or the selective use of defined *cis*-acting sequences [6]–[8]. Moreover, global enhancer sequences located outside the *Hox* clusters can coordinate the expression of several genes in a relatively promoter-unspecific manner [9]–[12]. Finally, large-scale chromatin remodeling events participate to the regulation of *Hox* loci [13], [14].

*Hox* RNAs and HOX proteins can colocalize, which reinforces the notion that transcriptional control is a primary mechanism for *Hox* gene regulation. However in some instances, HOX proteins are detected in a subdomain of the RNA pattern suggesting the existence of post-transcriptional control [8], [15]. The discovery of microRNAs that can mediate the targeted degradation of specific *Hox* transcripts has unveiled an additional level of regulation of *Hox* gene expression [16], [17]. In addition, antisense transcripts and long noncoding RNAs are found throughout *Hox* clusters and they are proposed to be part of the epigenetic regulation of *Hox* gene expression [18]–[22]. Altogether, these data indicate that a complex array of different modes of regulation is essential for the proper spatio-temporal *Hox* gene expression.

To fully understand the regulatory events governing *Hox* gene expression, we are using as a model the *Hoxa5* gene. This gene plays a crucial role during embryogenesis as well as being involved in tumorigenesis [23]–[25]. In the developing embryo, *Hoxa5* is expressed in the neural tube caudal to the posterior myelencephalon, in the axial skeleton up to the level of prevertebra (pv) 3 and in the mesenchymal component of several organs, including the trachea, the lung, the stomach, the intestine and the kidneys [26]–[31]. We have shown that the loss of *Hoxa5* function in the mouse affects a well-defined subset of structures mainly located at the cervico-thoracic level [23], [26], [27], [32], [33]. Aside from morphological defects in foregut derivatives and mammary glands [26], [29], [34], [35], the targeted disruption of the *Hoxa5* gene perturbs axial skeleton identity between pv3 and pv10, the anterior-most region of the *Hoxa5* domain of expression along the prevertebral axis [23], [27].

Polyadenylated transcripts of 1.8, 5.0, 9.5 and 11.0 kb in length encompassing *Hoxa5* coding sequences are produced in the embryo. They are also detected after birth in a tissue-specific fashion [23], [36]. The 1.8 kb transcript is the most abundant and it corresponds to the two characterized exons of the *Hoxa5* gene [37]. It encodes the 270 amino acid (a.a.) HOXA5 protein. Previous RNAase protection assays have shown that the larger forms initiate more upstream from sequences that remain to be identified [37]. Differences in the expression profile of these different transcripts are also observed: the 1.8 kb transcript is expressed as early as embryonic day (e) 8.0–8.25, whereas the larger transcripts are first detected around e8.5–8.75 [32]. The larger transcripts are present in more posterior structures of the embryo with an anterior limit of expression in the pv column corresponding to pv10, while that of the 1.8 kb transcript is pv3. In the neural tube, a posterior shift was observed for the larger transcripts [32]. Similarities between the *Hoxa7* expression profile and that of the larger transcripts indicate that they may share regulatory mechanisms [38].

The presence of multiple overlapping transcriptional units at the *Hoxa5* locus suggests that *Hoxa5* gene regulation may be complex. Using a transgenic approach, we have shown that several DNA control elements located both upstream and downstream the *Hoxa5* coding sequences are involved in the expression of the 1.8 kb transcript [32], [39]–[41]. An intricate situation prevails as some of these regulatory sequences are shared with the flanking *Hoxa4* gene, while others overlap with the *Hoxa6* coding sequences [32], [39], [42]. The presence of larger transcripts encompassing the *Hoxa5* coding sequences also implies that more DNA regions involved in *Hoxa5* gene regulation may be distributed along the cluster.

To assess the importance of the *Hoxa5* larger transcripts in *Hoxa5* gene function and regulation and to eventually define how they integrate in the developmental program, we have undertaken their molecular characterization. Our data revealed the complex organization of the different transcriptional units encompassing the *Hoxa5* and *Hoxa6* loci. It results from the use of three specific promoters and alternative splicing. Even though all these transcripts can potentially produce the genuine 270 a.a. HOXA5 protein, only the 1.8 kb form appears to generate the protein. Furthermore, the *Hoxa5* functional domain along the embryonic axis coincides with the expression region of the protein, where the larger transcripts are excluded. This pinpoints at the 1.8 kb form as the *Hoxa5* functional transcript in regional specification and leaves opened a role for the larger transcripts as long noncoding RNAs.

## Results

### Molecular characterization of the *Hoxa5* alternate transcripts

**Figure 1:**
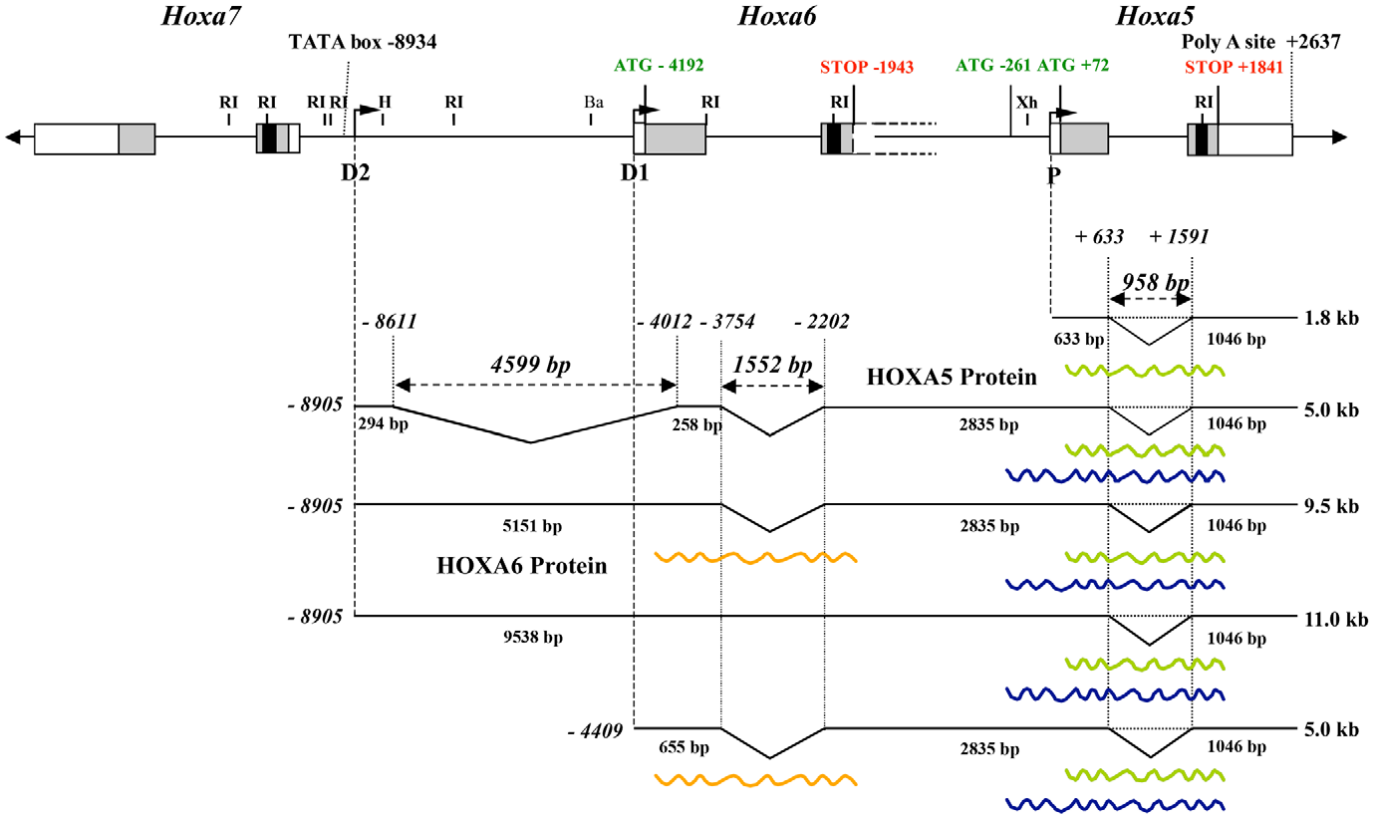
Schematic representation of the different transcripts encompassing *Hoxa5* sequences in the e12.5 mouse embryo. Genomic organization of the *Hoxa5*, *Hoxa6* and *Hoxa7* genes along the *HoxA* cluster. Black, grey and open boxes indicate homeobox, translated and transcribed sequences, respectively. The two known exons of *Hoxa5* and the two in-frame ATG are represented. Position +1 corresponds to the transcription initiation site of *Hoxa5* exon 1. The 3′ non-coding sequences of *Hoxa6* exon 2 extend further downstream into the *Hoxa6*-*Hoxa5* intergenic region and the adjacent *Hoxa5* coding sequences and they are indicated by dotted lines. The ATG of the putative HOXA6 protein is indicated. The promoters driving expression of the different transcripts are shown: proximal promoter, P; distal promoters D1 and D2. The transcripts are represented underneath based on northern, 5′ RACE, 3′-RACE and RT-PCR assays used to define their molecular structure. *Hoxa5* intron is represented by a dotted line to indicate the non-spliced isoforms. The longest ORFs deduced from the sequence of each transcript are represented by waved lines: the 270 a.a. HOXA5 protein, the 381 a.a. HOXA5 isoform and the HOXA6 protein. Ba, *Bam*HI; H, *Hind*III; RI, *Eco*RI; Xh, *Xho*I.

Previous northern analysis of polyA^+^ RNA from mouse embryo using a DNA probe corresponding to the 3′-untranslated region of the second exon of the *Hoxa5* gene has shown that polyadenylated transcripts of approximately 1.8, 5.0, 9.5 and 11.0 kb in length contain sequences from the *Hoxa5* locus [23]. The 1.8 kb transcript corresponds to the putative *Hoxa5* transcript as demonstrated from cDNA sequence analyses [36], [37]. We thus aimed to determine the molecular origin of the larger transcripts. To do so, we applied a series of molecular approaches, and by merging all the data obtained from northern, 3′- and 5′-RACE, RT-PCR and cDNA analyses, we established a schematic representation of the major *Hoxa5* transcripts produced in the e12.5 mouse embryo (Fig. 1).

**Figure 2:**
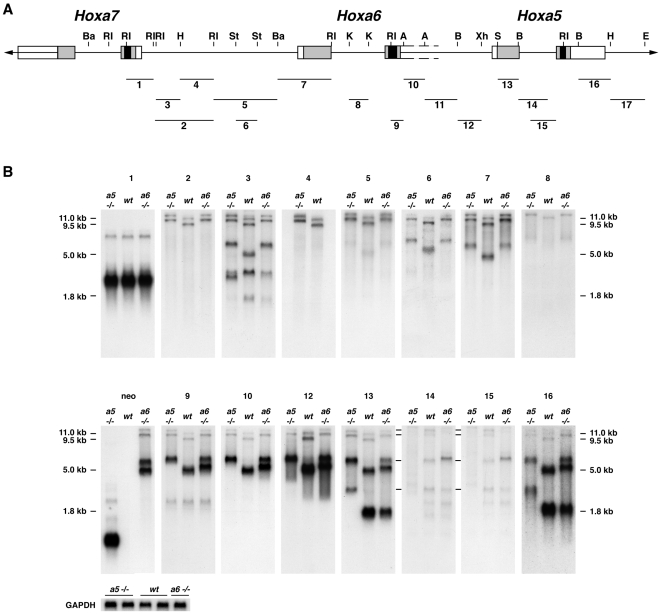
Molecular characterization of *Hoxa5* transcripts by northern analysis. (A) Genomic organization of the *Hoxa5*, *Hoxa6* and *Hoxa7* genes along the cluster. Black, grey and open boxes indicate homeobox, translated, and transcribed sequences, respectively. Probes used for northern analyses are indicated by numbered lines (1–17). (B) Northern analyses of polyA^+^ RNA from e12.5 wild-type, *Hoxa5* ^−/−^ and *Hoxa6* ^−/−^ mouse embryos were performed with probes covering the genomic region located between *Hoxa5* and *Hoxa7*. The mutated form of the *Hoxa5* transcripts produced in *Hoxa5* ^−/−^ mice are 1 kb larger than the endogenous ones due to the insertion of the *neo* cassette into the homeobox sequence. A similar situation prevails for the *Hoxa6* mutation where the insertion of the *neo* cassette in the *Hoxa6* homeobox sequence also disrupts all transcripts encompassing the *Hoxa5* and *Hoxa6* loci, except the 1.8 kb form. The 5.0, 9.5 and 11.0 kb transcripts contain *Hoxa5* exons 1 and 2 sequences but initiated further upstream of the known 1.8 kb transcript start site in the *Hoxa6*-*Hoxa7* intergenic region. The difference in length between the 9.5 and 11.0 kb transcripts is due to the *Hoxa6* intron sequences present only in the 11.0 kb transcrit (probe 8). All transcripts end at the same polyadenylation site at the 3′ end of *Hoxa5* exon 2 since no hybridization was observed by northern analyses with probes covering more than 1 kb of genomic sequences 3′ to the *Hoxa5* polyA site (probe 17; data not shown). The lines on the left of northern blots 14 and 15 indicate *Hoxa5* non-spliced isoforms that are 1 kb larger. *Gapdh* was used as a loading control. A, *Acc*I; B, *Bgl*II; Ba, *Bam*HI; E, *Eag*I; H, *Hind*III; K, *Kpn*I; RI, *Eco*RI; S, *Sac*I; St, *Stu*I; Xh, *Xho*I.

First, we performed northern analyses with e12.5 mouse embryo polyA^+^ RNA using as antisense riboprobes several genomic fragments encompassing *Hoxa5* and flanking *Hox* genes (Fig. 2). The RNA was obtained from wild-type (wt), *Hoxa5* ^−/−^ and *Hoxa6* ^−/−^ embryos. We took advantage of the mutant forms of the *Hoxa5* and *Hoxa6* transcripts produced in *Hoxa5* ^−/−^ and *Hoxa6* ^−/−^ mice, respectively. These mutant transcripts are 1 kb larger than the endogenous ones due to the insertion of a 1 kb *neo* cassette into the homeobox sequence of each gene [23], [43], [44]. This difference in length allowed us to distinguish the alternate *Hoxa5* transcripts among the several products detected. Northern analyses of polyA^+^ RNA from wt and *Hoxa5* ^−/−^ embryos revealed that the 1.8, 5.0, 9.5 and 11.0 kb transcripts contained the two *Hoxa5* exons and they were all affected by the insertion of the *neo* cassette into the *Hoxa5* mutant allele as previously shown (probes 13 and 16; Fig. 2) [23]. Moreover, these sense transcripts were all transcribed from the same DNA strand. The 1.8 kb transcript corresponded to the two *Hoxa5* exons. As shown by probes 2 to 12, the 5.0, 9.5 and 11.0 kb RNA species initiated in the *Hoxa6*-*Hoxa7* intergenic region further upstream from the identified 1.8 kb transcript start site (position +1; Figs. 1 and 2) [37]. These larger transcripts contained the *Hoxa6* sequences and they showed the expected shift in size in *Hoxa6* ^−/−^ RNA due to the presence of the *neo* cassette [44]. A complex splicing pattern also prevailed explaining the difference in length between the 5.0, 9.5 and 11.0 kb transcripts. The *Hoxa6* intron sequences were only detected in the 11.0 kb transcript (probe 8; Fig. 2), while some *Hoxa6*-*Hoxa7* intergenic sequences did not hybridize to the 5.0 kb band (probes 2, 4 and 5; Fig. 2). Faint bands approximately 1 kb larger than the expected transcripts were also distinguished in the wt specimens with probes 14 and 15 that correspond to *Hoxa5* intron sequences, suggesting that transcripts with unspliced *Hoxa5* intron sequences may exist at low abundance (Fig. 2). Additional bands of about 2.5 kb in length were detected with the *Hoxa6*-*Hoxa7* intergenic probe 3. Their origin was not investigated but they could correspond to RNA species initiating in the *Hoxa6*-*Hoxa7* intergenic region that skip the *Hoxa6* and *Hoxa5* loci to continue further downstream in the *Hoxa4*-*Hoxa5* sequence, like the GenBank mRNA AK051552, or extend towards the vicinity of the *Hoxa3* gene, as the Y11717 mRNA (GenBank). Finally, all 1.8, 5.0, 9.5 and 11.0 kb transcripts ended at the same polyA site at the 3′ end of *Hoxa5* exon 2 (position +2637) as revealed by 3′-RACE (Rapid Amplification of cDNA Ends) experiments. This was also demonstrated by the lack of hybridization in northern analysis with a riboprobe located in genomic sequences 3′ to the *Hoxa5* polyA site (Fig. 2; data not shown).

To map the transcriptional start site of the larger *Hoxa5* transcripts, we designed a 5′-RACE strategy based on the data obtained from the northern analyses. We used different sets of primers specific either to the 9.5 and 11.0 kb transcripts (primer 1) or located in sequences shared by the 5.0, 9.5 and 11.0 kb transcripts (primers 2 and 3; Fig. S1A). Clones obtained with primer 1 indicated that the 9.5 and 11.0 kb transcripts initiate in the *Hoxa6*-*Hoxa7* intergenic region at position –8905 bp, which is about 2.3 kb downstream of the 3′-end of *Hoxa7* gene. With primers 2 and 3, 5′-RACE products revealed the presence of a 4.6 kb intron and an initiation site coinciding with that of the largest transcripts at position –8905 bp. A second population of clones was also obtained with primer 2 with a transcription start site at position –4409 bp, which corresponds to the putative first base of *Hoxa6* exon 1. Thus, two distal promoters, one related to the *Hoxa6* gene (promoter D1) and a more distal one located dowstream the *Hoxa7* gene (promoter D2), participate in the production of the *Hoxa5* alternate transcripts (Fig. 1).

Finally to resolve the molecular structure of the different transcripts, we used various combinations of primers in RT-PCR experiments (Fig. S1B). Sequencing data of the clones obtained confirmed the importance of alternative splicing in the production of the various *Hoxa5* transcripts and revealed other minor forms (clones pLJ282 and 284).

Figure 1 summarizes the molecular characterization of the different *Hoxa5* transcripts and from this, several observations were made. First, the sequences between the *Hoxa7* and the *Hoxa5* genes can be entirely transcribed to give rise to the 11.0 kb transcript. Second, the 5.0 kb band detected by northern analyses included two main RNA species, one initiating at position –8905 bp, like the larger forms of 9.5 and 11.0 kb, and containing a large intron of 4.6 kb (identified as the 5 kb-*Hoxa5* transcript), and a second starting at –4409 bp, from the putative *Hoxa6* promoter (identified as the 5 kb-*Hoxa6/a5* transcript). Third, no specific *Hoxa6* transcript corresponding solely to the two known *Hoxa6* exons was detected by northern analysis. Indeed, such signal was not observed with a probe including the putative *Hoxa6* exon 1 sequences (probe 7; Fig. 2). A weak band of about 2.4 kb in length was seen with the *Hoxa6* exon 2 probe containing part of the homeobox sequence (probe 9; Fig. 2). However, it could not correspond to a *Hoxa6* transcript since it did not produce a mutant form 1 kb larger in the *Hoxa6* ^−/−^ RNA sample. Sequence blast of probe 9 against the mouse genome revealed homologies with some *Hox* genes, the highest being 94% homology with the *Hoxa7* homeobox sequence (data not shown). Since the 2.4 kb band matched the main transcript seen with probe 1, which included *Hoxa7* homeobox sequence, it is likely that this faint band may result from the cross-hybridization of probe 9 with the major *Hoxa7* transcript.

Our northern and 3′-RACE studies unveiled that all *Hoxa5* transcripts use the polyA site of the *Hoxa5* gene. Search for polyadenylation sequences at the *Hoxa6* locus did not reveal the presence of a consensus site nearby the presumptive 3′-end of the *Hoxa6* gene. Identified consensus motifs were either overlapping the end of the *Hoxa6* homeobox sequence (position −2006 bp relative to the start site of the 1.8 kb transcript), or located further downstream in the *Hoxa6*-*Hoxa5* intergenic region (positions −825 bp and −468 bp). The lack of a functional polyadenylation sequence at the *Hoxa6* locus was further confirmed by the presence of a *neo* transcript in *Hoxa6* ^−/−^ RNA sample of about 5.5 kb in length (probe neo; Fig. 2). This transcript initiated at the *MC1* promoter of the *MC1neo* cassette, which does not contained a polyA addition signal [44]. Thus, transcription of the *neo* cassette must end at the nearest functional polyadenylation site, the latter being localized 3′ of the *Hoxa5* gene. In summary, the use of different promoters and alternative splicing may account for the production of several transcriptional units containing sequences from both *Hoxa5* and *Hoxa6* loci.

### Transcriptional activity in the *Hoxa6*-*Hoxa7* intergenic region

**Figure 3:**
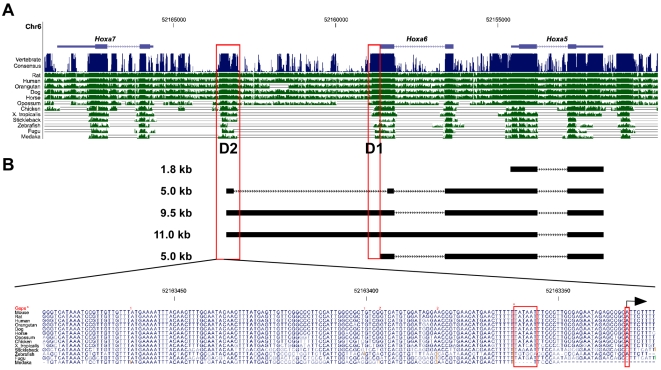
Evolutionary conservation of the *Hoxa5-Hoxa7* genomic region among animal species. (A) The mouse sequence of the *Hoxa5-Hoxa7* loci was compared to that of rat, human, orangutan, dog, horse, opossum, chicken, xenopus tropicalis, stickleback, zebrafish, fugu and medaka using the UCSC genome browser. The regions with vertical lines indicate conserved sequences. In addition to *Hox* exons, the DNA regions located upstream the *Hoxa5* distal transcription start sites at positions −4409 bp (D1) and −8905 bp (D2) show high homology between divergent species (boxes), suggesting the presence of evolutionary conserved important regulatory DNA elements. (B) Alignment of the nucleotides of a 160-bp DNA fragment from the D2 region indicates the presence of a consensus TATA box and a transcription initiation site (boxes) in most species.

Our observation that the 5 kb-*Hoxa5*, 9.5 and 11.0 kb alternate transcripts initiated from a DNA region located downstream the *Hoxa7* gene prompted us to define the transcriptional activity of the sequences encompassing the potential distal promoters D1 and D2. We first performed comparison of the sequences encompassing the *Hoxa5*, *Hoxa6* and *Hoxa7* loci between divergent vertebrate species using the University of California, Santa Cruz (UCSC) Genome Browser (http://genome.ucsc.edu/; Mouse July 2007 assembly) [45]. As expected, *Hox* exon sequences showed very high homology (Fig. 3). A ∼500 bp DNA region surrounding the transcription start site at position −8905 bp and the D2 promoter was also highly conserved among the species. Alignment of the nucleotide sequences indicated a DNA region of 160 bp, which includes a putative TATA box at position −8934 bp, very highly preserved, arguing for the presence of evolutionary conserved important regulatory DNA elements that may be involved in the production of the larger transcripts. Highly homologous sequences located just 5′ from *Hoxa6* exon 1 and corresponding to the putative D1 promoter were also found.

**Figure 4:**
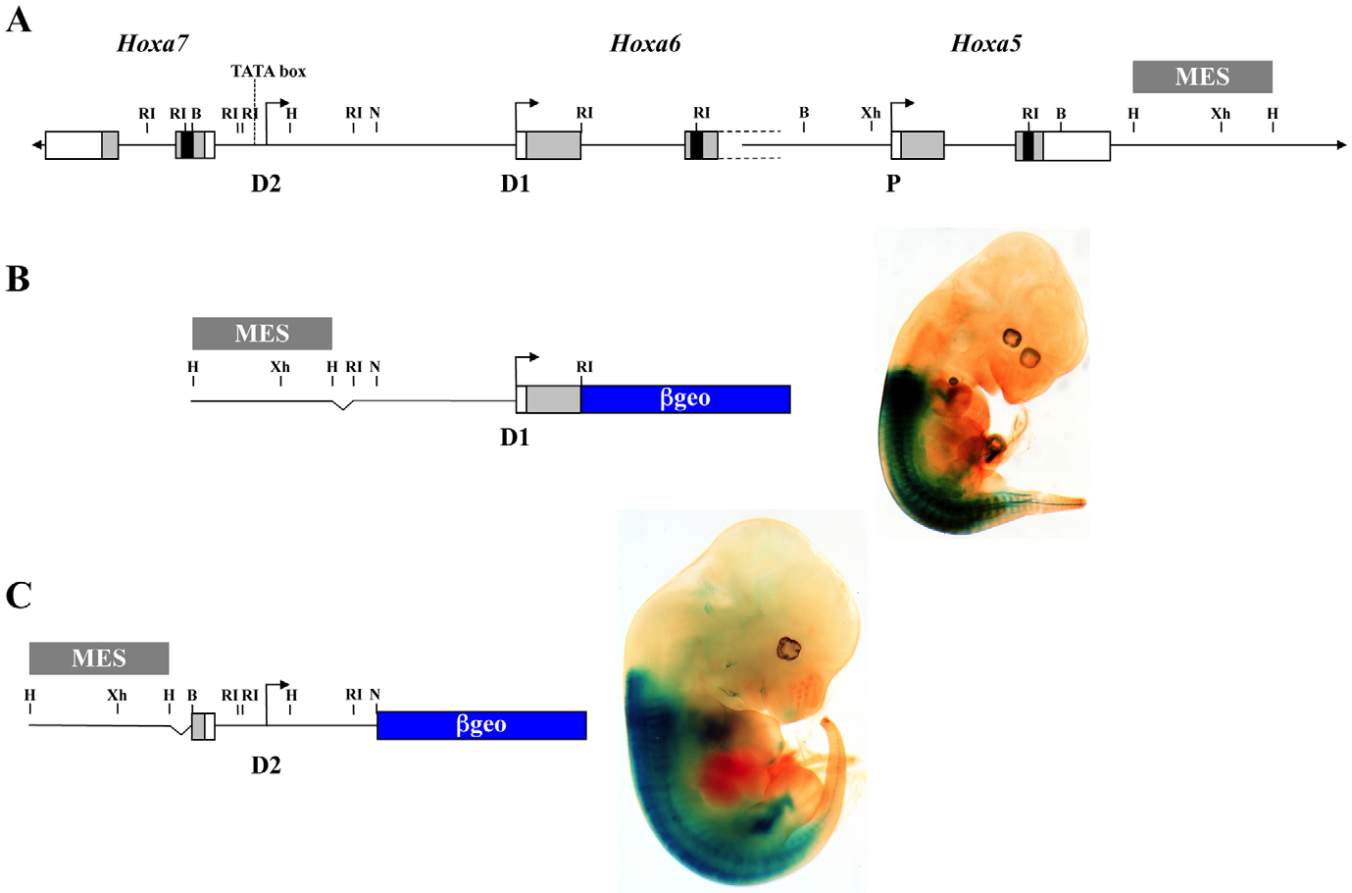
Transcriptional activity of the D1 and D2 putative promoters in e12.5 transgenic embryos. (A) A schematic representation of the *Hoxa5*, *Hoxa6* and *Hoxa7* genes along the *HoxA* cluster. The proximal promoter P and the two distal promoters D1 and D2 are indicated. (B) Detection of β-galactosidase activity in *D1-lacZ* transgenic embryos in presence of the mesodermal enhancer sequence (MES) indicates that the 4 kb-DNA region encompassing the putative *Hoxa6* promoter (D1) can drive *Hox*-like expression along the antero-posterior axis. (C) As well, a 4 kb-DNA fragment containing the D2 putative promoter region of the larger transcripts possesses a similar transcriptional activity. B, *Bgl*II; H, *Hind*III; N, *Nru*I; RI, *Eco*RI; Xh, *Xho*I.

The transcriptional activity of the distal promoters D1 and D2 was directly assessed by transgenesis (Fig. 4). For each promoter, a ∼4 kb DNA fragment containing sequences flanking the transcription start site was fused to an IRES-βgeo cassette used as a reporter. A MES enhancer sequence, known to drive *Hoxa5* regionalized expression along the embryonic axis, was added to each construct in order to improve the detection of a minimal promoter activity [32]. A *Hox*-like staining pattern was observed with the two transgenes indicating that the sequences upstream the distal transcription initiation sites D1 and D2 possess promoter activity.

### Differential expression pattern of the *Hoxa5* transcripts

**Figure 5:**
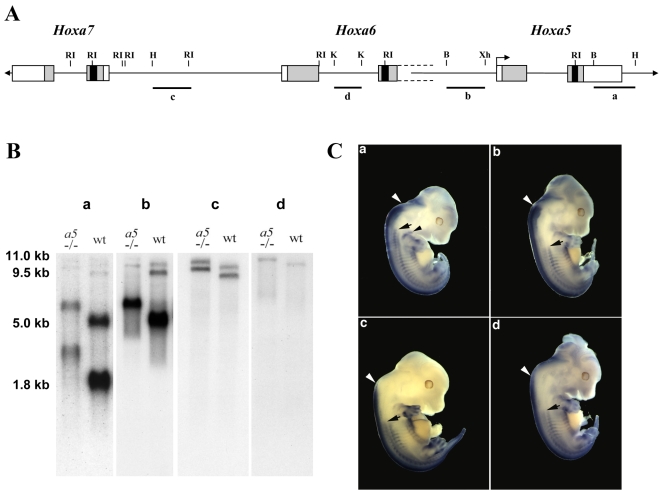
Differential expression pattern of *Hoxa5* transcripts. (A) Genomic organization of the *Hoxa5*, *Hoxa6* and *Hoxa7* genes along the cluster. Probes a, b, c and d used for northern blot analyses and whole-mount *in situ* hybridization are indicated below. (B) Northern blots of polyA^+^ RNA extracted from e12.5 wild-type and *Hoxa5* ^−/−^ embryos were hybridized with each probe. (C) Whole-mount *in situ* hybridization of e12.5 wild-type embryos with probes a-d. Anterior limits of expression are indicated for the neural tube (white arrowheads) and the prevertebral column (black arrows). The different profiles reveal the specific expression of the transcripts: probe a allows to identify the structures that exclusively express the 1.8 kb transcript. Theses structures include the pv3-pv10 axial domain and the pectoral girdle (black arrowhead). The larger transcripts are expressed in more posterior structures than the 1.8 kb transcript. B, *Bgl*II; H, *Hind*III; K, *Kpn*I; RI, *Eco*RI; Xh, *Xho*I.

Our previous studies have demonstrated that the 1.8 kb transcript is expressed earlier during embryogenesis and in more anterior structures than the larger transcripts [32]. To gain information on the potential function of the alternate transcripts during embryogenesis, we performed comparative whole-mount *in situ* hybridization analyses at e12.5. Since all transcripts included the two *Hoxa5* exons corresponding to the 1.8 kb transcript and shared most of their sequences, we used probes that recognize either all transcripts or combinations of the larger forms. As shown on figure 5A and B, probe “a” contains *Hoxa5* exon 2 sequences common to all transcripts; probe “b” corresponds to the *Hoxa5*-*Hoxa6* intergenic region recognizing the 5.0, 9.5 and 11.0 kb forms; probe “c” is localized in the *Hoxa6*-*Hoxa7* intergenic region and detects the 9.5 and 11.0 kb transcripts; and probe “d” includes *Hoxa6* intron sequences hybridizing only to the 11.0 kb transcript. The expression profile detected with probe “a”, but not with probes “b”, “c” and “d”, revealed structures that exclusively express the 1.8 kb transcript (Fig. 5C). In the pv column, the anterior limit of expression of the 1.8 kb transcript corresponded to pv3, while the 5.0, 9.5 and 11.0 transcripts shared the same boundary at pv10. In the neural tube, a posterior shift was observed for the larger transcripts, and the shift was more caudal with probes “c” and “d”. In the future pectoral girdle, hybridization signal was detected only with probe “a”. Previous *in situ* hybridization experiments on e12.5 embryo sections have shown a strong expression with probe “a” in the mesenchymal component along the entire respiratory tract while probe “b” produced a weak signal restricted to the distal tip of the lungs [26], [27]. Similarly, hybridization in the thyroid gland region was observed only with probe “a” (data not shown) [35]. In the developing gastrointestinal tract, a dynamic *Hoxa5* expression pattern prevails. Probe “a” detected expression in the gut mesenchyme as early as e9.0, while the onset of expression with probe “b” was delayed to e12.5 in the foregut, and e10.5 in the midgut [28], [29]. In the hindgut, expression of the larger transcripts was detected as early as e9.5 indicating that they shared the same onset as the 1.8 kb transcript (Fig. S2). After e12.5, the expression profile in the developing stomach was similar for probes “a” and “b” with a widespread distribution throughout the gastric mesenchyme until e17.5, followed by restriction to the submucosa and muscular layers and extinction at postnatal day 15 (data not shown) [29]. In the mid- and hindgut, expression was detected with both probes in the mesenchyme up to e14.5 and e17.5, respectively. Expression of the larger transcripts then extinguished in the midgut and the hindgut, while that of the 1.8 kb transcript got restricted to the enteric nervous system and was maintained after birth (Fig. S2) [28].

**Figure 6:**
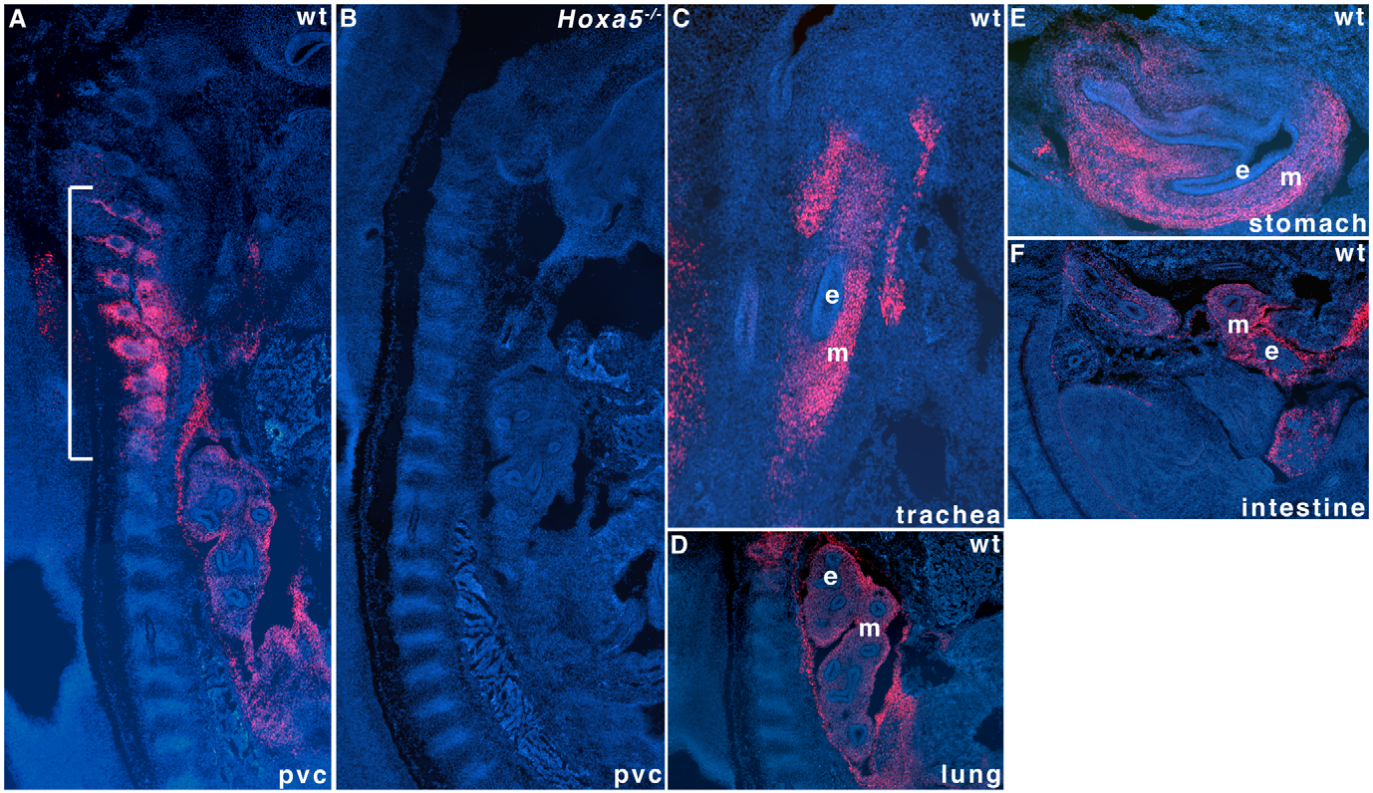
Restricted spatial distribution of HOXA5 protein along the antero-posterior axis and in the respiratory and digestive tracts. HOXA5 immunoreactivity is detected in the pv3-pv10 region of the prevertebral column (pvc) of e12.5 wild-type mouse embryos by immunofluorescence (bracket; A). No immunoreactivity is seen in the *Hoxa5* ^−/−^ specimens confirming the absence of the protein in the null mutant mouse line (B). HOXA5 is also detected in the mesenchymal component of the trachea (C), lung (D), stomach (E) and intestine (F) of e12.5 mouse embryo. e, epithelium; m, mesenchyme.

The differential expression of the transcripts encompassing *Hoxa5* sequences along the antero-posterior axis of the skeleton and the rostro-caudal axis of the developing gut reflects the organization of the different promoters (P, D1 and D2) along the cluster and respects the relationship of colinearity characterizing the *Hox* complexes. It also raises questions about the role played by each of these transcripts during development. Even though the *Hoxa5* mutation perturbs all *Hoxa5* transcripts, most of the defects observed in the *Hoxa5* ^−/−^ mutant mice are confined to the cervico-thoracic region and they affect structures and organs that solely express the 1.8 kb transcript. Using a HOXA5-specific antibody, we looked at the HOXA5 protein distribution in the e12.5 mouse embryo [46]. HOXA5 immunoreactivity was observed along the pv column in the pv3-pv10 region and in the mesenchyme of the trachea, lung, stomach and intestine (Fig. 6A, C–F). No immunostaining was detected in *Hoxa5* ^−/−^ specimens (Fig. 6B). Except for the gastrointestinal tract where all *Hoxa5* transcripts were detected (Fig. S2), the expression seen in the pv column and the respiratory tract matched that of the 1.8 kb transcript, raising the possibility that only the 1.8 kb transcript produces the HOXA5 protein.

### Translational capability of the *Hoxa5* transcripts

There are multiple ORFs predicted from the sequence of the different *Hoxa5* transcripts and the longest ones are represented in figure 1. All *Hoxa5* transcripts include the HOXA5 ORF suggesting that they can potentially produce the genuine 270 a.a. HOXA5 protein. Sequence analysis also revealed the presence of a distal in-frame ATG codon located 333 nucleotides upstream of the proximal promoter (P; Fig. 1) that can produce a larger HOXA5 isoform of 381 a.a. Moreover, a HOXA6 protein of 232 a.a. can potentially be translated from the 5 kb-*Hoxa6/a5* and the 9.5 kb transcripts, raising the possibility of bicistronic transcriptional units.

**Figure 7:**
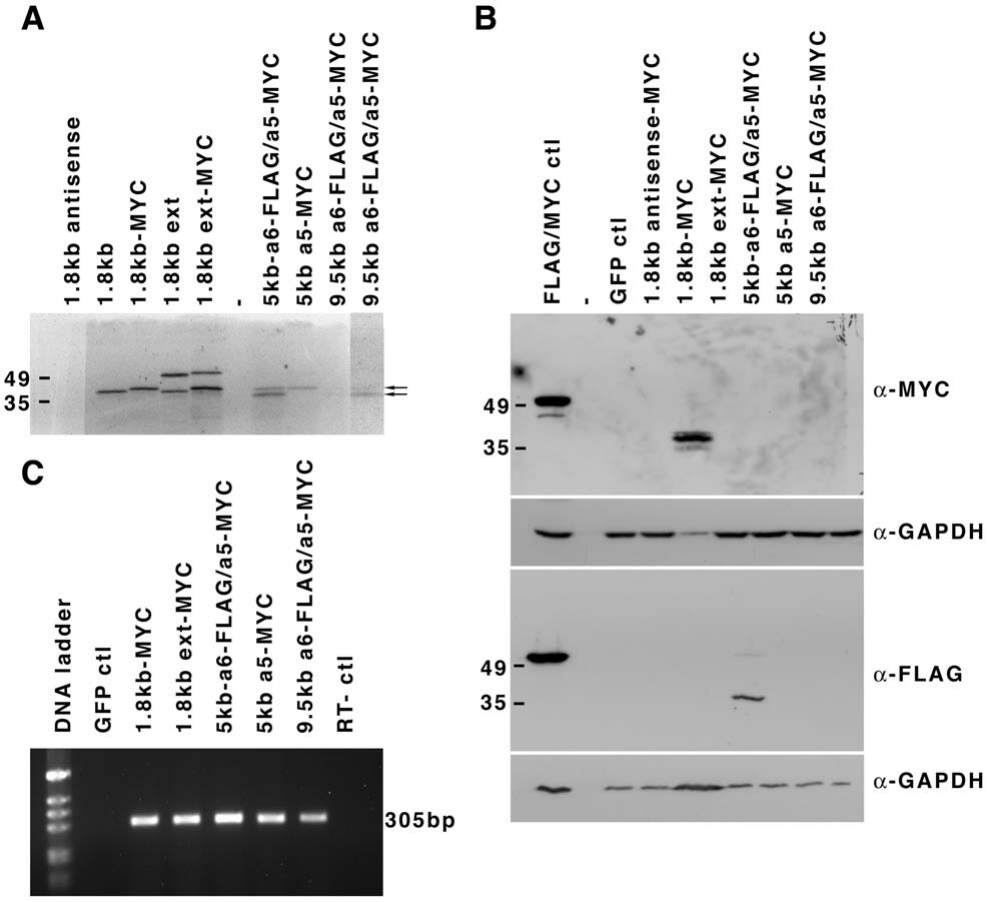
HOXA5 protein production from *Hoxa5* transcripts. (A) Expression vectors carrying cDNAs corresponding to the 1.8 kb (with and without a MYC-tag), the extended 1.8 kb (MYC-tagged and non-tagged), the 5.0 kb-*Hoxa6*-FLAG/*a5*-MYC, the 5.0 kb-*Hoxa5*-MYC and the 9.5 kb *Hoxa6*-FLAG/*a5*-MYC transcripts were tested *in vitro* using a coupled transcription/translation system. A [S^35^]-radiolabeled protein corresponding to a ∼38 kD genuine HOXA5 protein is translated from all vectors. The larger HOXA5 isoform of ∼50 kD is only translated from the extended 1.8 kb cDNA version. The 5.0 kb-*Hoxa6*-FLAG/*a5*-MYC and the 9.5 kb *Hoxa6*-FLAG/*a5*-MYC vectors also produce a ∼36 kD band likely corresponding to the putative HOXA6 protein. Translation of the HOXA5 and HOXA6 proteins from the 9.5 kb *Hoxa6*-FLAG/*a5*-MYC vector is weakly detected and a longer exposure is shown with arrows to indicate the position of both proteins. (B) The HEK293 cells were transfected with the MYC-tagged version of the 1.8 kb and extended 1.8 kb expression vectors and with the 5.0 kb-*Hoxa6*-FLAG/*a5*-MYC, the 5.0 kb-*Hoxa5*-MYC and the 9.5 kb *Hoxa6*-FLAG/*a5*-MYC vectors. In parallel, control plasmids expressing either the green fluorescent protein (GFP ctl), the 1.8 kb *Hoxa5*-MYC in the antisense orientation or the pMEK1-MYC-FLAG plasmid (MYC/FLAG ctl) were transfected. Protein lysates were western-blotted with anti-MYC or anti-FLAG antibodies. Solely the 1.8 kb-MYC vector produces a genuine HOXA5 protein whereas the HOXA6 protein is only detected with the 5.0 kb-*Hoxa6*-FLAG/*a5*-MYC plasmid. GAPDH was used as a loading control. (C) RNA expression of each expression vector transfected in HEK293 cells was tested by RT-PCR. A 305 bp fragment is observed in RNA samples from HEK293 cells transfected with the *Hoxa5* cDNA vectors. No expression is detected in the GFP ctl specimen.

To define the capacity of the larger transcripts to produce the HOXA5 protein, we made expression vectors containing the entire 5 kb-*Hoxa5*, 5 kb-*Hoxa6/a5* or 9.5 kb cDNA sequence with a MYC tag at the carboxy terminus of the HOXA5 protein. We also added a FLAG tag at the carboxy terminus of the HOXA6 protein for the 5 kb-*Hoxa6/a5* and 9.5 kb cDNA vectors. As a control, we used a MYC-tagged version of the 1.8 kb cDNA. In addition, we made a vector containing an extended version of the 1.8 kb transcript (up to position -555 relative to the start site of the 1.8 kb transcript) that includes the distal ATG codon. The plasmids were first tested *in vitro* using a coupled transcription/translation assay with incorporation of radio-labeled methionine (Fig. 7A). As expected, vectors containing the 1.8 kb cDNA sequence with or without the MYC tag produced radioactive products of about 38 kD in size, slightly larger for the vector carrying the MYC tag and which corresponded to the 270 a.a. HOXA5 protein. For the extended version of the 1.8 kb transcript, the 38 kD HOXA5 protein was produced as well as a protein of about 50 kD, compatible to the 381 a.a. isoform. For the vector carrying the 5 kb-*Hoxa5* cDNA, the HOXA5-MYC isoform of 270 a.a. was the unique band observed. In the case of the vector containing the 5 kb-*Hoxa6/a5* cDNA sequence, the 270 a.a. HOXA5-MYC protein was detected as well as a smaller protein of about 36 kD, likely corresponding to the HOXA6-FLAG protein. Similar observations were made for the 9.5 kb cDNA, even though the two bands were faint. No band related to the larger HOXA5 isoform was detected with the 5 kb-*Hoxa5*, the 5 kb-*Hoxa6/a5* and the 9.5 kb cDNAs. Thus when tested *in vitro*, all vectors can produce the genuine HOXA5 protein. The 5 kb-*Hoxa6/a5* and 9.5 kb transcripts can also generate the HOXA6 protein, acting as bicistronic units.

Transfection assays in HEK293 cells followed by western analyses with the MYC or FLAG antibodies showed that only the 1.8 kb-MYC vector produces the 270 a.a. HOXA5-MYC protein (Fig. 7B). The HOXA5-MYC isoform of 381 a.a. was neither produced from the extended version of the 1.8 kb transcript nor from the 5 kb-*Hoxa5*, the 5 kb-*Hoxa6/a5* and the 9.5 kb vectors. Moreover, only the 5 kb-*Hoxa6/a5* vector produced the HOXA6-FLAG protein of 232 a.a. As verified by RT-PCR analysis, all *Hoxa5* cDNA expression vectors were transcribed in HEK293 cells (Fig. 7C). In summary, all *Hoxa5* transcripts can be efficiently translated in *in vitro* assays. However in cell cultures, only the 1.8 kb transcript can encode the 270 a.a. HOXA5 protein and the HOXA6 protein can solely be produced from the 5 kb-*Hoxa6/a5* transcript.

### Skeletal transformations in *Hoxa6* and *Hoxa5*; *Hoxa6* transheterozygous mutant mice

The *Hoxa6* mutant mouse line provides a valuable tool for investigating the role of the larger *Hoxa5* transcripts since the insertion of the *MC1neo* cassette into the *Hoxa6* homeobox sequences disrupts the 5.0, 9.5 and 11.0 RNA species encompassing the *Hoxa5* and *Hoxa6* loci (Fig. 2). The *Hoxa6* mutation causes a relatively mild phenotype, which consists in the presence of ectopic ribs on the 7th cervical vertebra (C7) in less than half of the mutants [44]. One puzzling aspect of the *Hoxa6* ^−/−^ phenotype is that it is incompatible with the pv10 anterior expression boundary of the gene, as established by the expression analysis using probes specific for the larger transcripts (Fig. 5C). Two possibilities could account for this discrepancy. The integrity of the larger transcripts including *Hoxa6* sequences is necessary for the correct patterning at the pv7 axial level. Alternatively, the presence of the *neo* cassette in the *Hoxa6* locus may interfere with the expression of the nearby *Hoxa5* gene, which then can impact on the skeletal phenotype. Ectopic ribs on C7 are a hallmark of the *Hoxa5* mutation, as they are found in most *Hoxa5* mutants [23], [27]. Moreover, transcriptional interference is not unusual in *Hox* mutations, and we have previously reported the deleterious long-range *cis* effect of the *Hoxa4* mutation on *Hoxa5* expression [27]. To discriminate between these options and to define the respective role of the *Hoxa5* and *Hoxa6* genes in the specification of the cervico-upper thoracic region, we generated *Hoxa5*; *Hoxa6* transheterozygous animals (*Hoxa5* ^+/−^; *Hoxa6* ^−/+^), which are heterozygotes for both genes on different chromosomes. First, we examined the skeleton of new cohorts of single mutants and transheterozygous newborn pups (Table 1). In this mixed genetic background, *Hoxa5* ^−/−^ mutants displayed the skeletal transformations previously reported: the lack of tuberculum anterior on C6 in 84% of the specimens analyzed; the presence of ectopic ribs on C7 (87%), most being present on both sides of the vertebra; abnormal acromion (42%) and fused tracheal rings (100%). *Hoxa5* ^+/−^ pups also presented the C7 homeotic transformation at a lesser frequency (57%) than the *Hoxa5* ^−/−^ mutants, but with a much higher incidence than the *Hoxa6* ^−/−^ mutants (35%; Table 1). Ectopic ribs on C7 were observed in 58% of the *Hoxa5* ^+/−^; *Hoxa6* ^−/+^ pups analyzed, a frequency similar to that of *Hoxa5* ^+/−^ mutants, suggesting that the *Hoxa6* contribution to the C7 skeletal specification was weak.

**Table 1:** Newborn skeletal morphology according to the *Hoxa5* and *Hoxa6* genotypes.

	Genotype					
	wt	*Hoxa5* ^+/−^ *Hoxa6* ^+/+^	*Hoxa5* ^−/−^ *Hoxa6* ^+/+^	*Hoxa5* ^+/−^Hoxa6^−/+^	*Hoxa5* ^+/+^ *Hoxa6* ^+/−^	*Hoxa5* ^+/+^ *Hoxa6* ^−/−^
Tuberculum anterior on C6 a						
Absent	-	-	32	-	-	-
Present	14	44	6	38	20	26
Ribs on C7^a^						
Absent	12	16	5	16	7	17
Present	2	28	33	22	13	9
Unilateral	-	6	3	6	1	5
Bilateral	1	11	15	8	6	2
Acromion^a^						
Normal	14	44	22	38	19	26
Abnormal	-	-	16	-	1	-
Trachea						
Normal	7	22	-	19	10	13
Abnormal	-	-	19	-	-	-
Number of animals	7	22	19	19	10	13

a left and right sides were scored independently.

**Figure 8:**
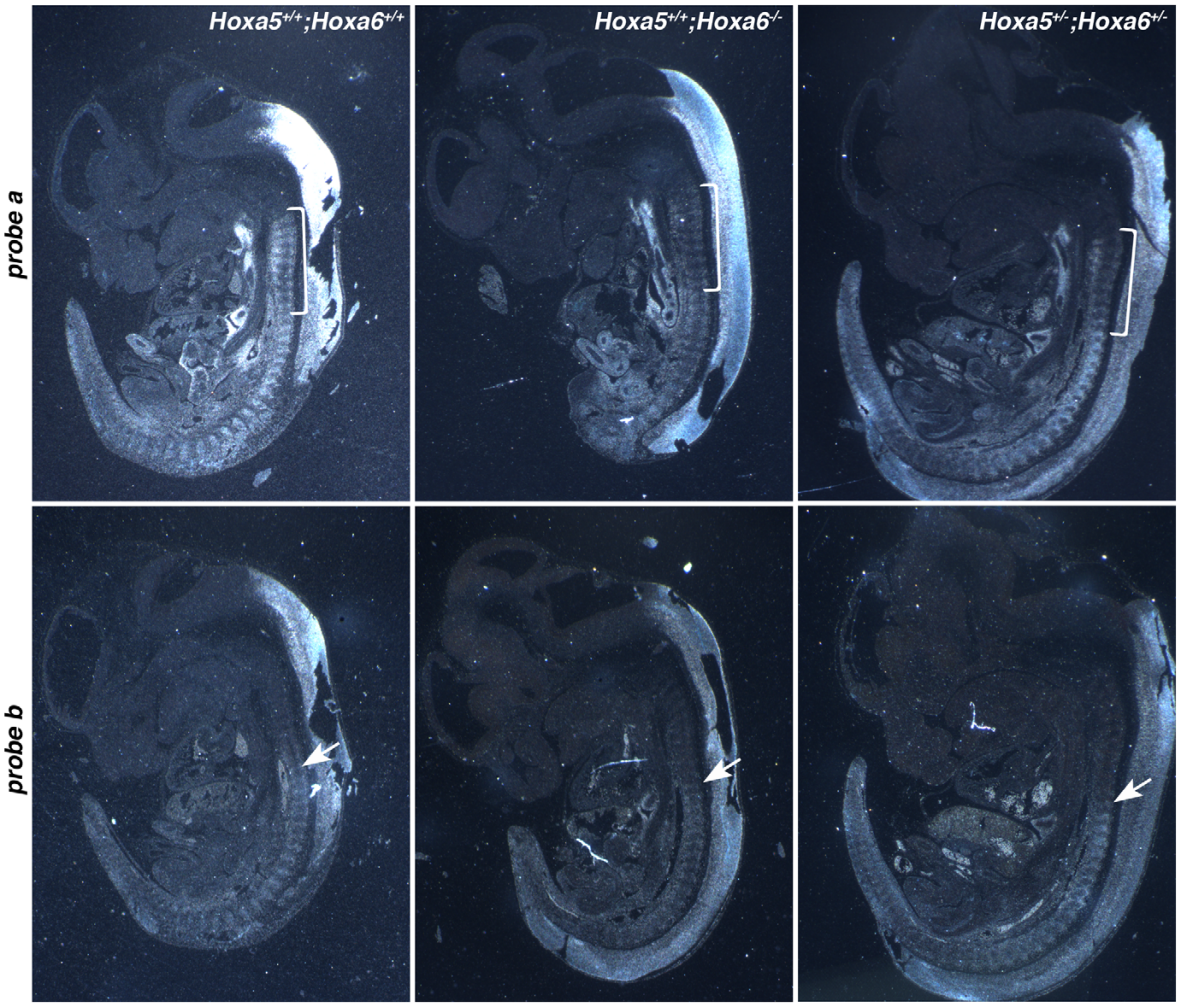
Comparative expression patterns of *Hoxa5* transcripts in e12.5 wild-type, *Hoxa6* homozygous and *Hoxa5*; *Hoxa6* transheterozygous mutants. *In situ* hybridization experiments were performed on comparable sagittal sections. Representative specimens are shown. Genotype is indicated on the top right of each column and probe on the left of each row. The bracket indicates the pv3-pv10 domain, which hybridizes with probe a in wild-type specimens, but not with probe b. In *Hoxa6* ^−/−^ and *Hoxa5* ^+/−^; *Hoxa6* ^−/+^ mutants, the signal in this region is significantly decreased with probe a. With probe b, the anterior limit of expression corresponds to pv10 in all samples regardless of the genotype (arrows) with no major change in signal intensity.

We also monitored *Hoxa5* expression in *Hoxa6* ^−/−^ and *Hoxa5* ^+/−^; *Hoxa6* ^+/−^ e12.5 embryos by *in situ* hybridization using the riboprobes “a” and “b” described above (Figs. 5A and 8). The signal detected with probe “a” was specifically less intense in the pv3-pv10 region of the pv column from *Hoxa6* ^−/−^ and *Hoxa5* ^+/−^; *Hoxa6* ^+/−^ embryos when compared to wt specimens. No change in expression was observed with probe “b”. Thus, the disruption of the large transcripts by the *Hoxa6* mutation does not have a major impact on axial specification as no skeletal anomaly was seen for the vertebrae localized caudally of pv10. Moreover, the *Hoxa6* mutation alters expression of the 1.8 kb transcript in the pv3-pv10 region. One possible explanation may be that the insertion of the *neo* cassette into the *Hoxa6* locus impairs the activity of the *Hoxa5* proximal promoter, which consequently alters the specification of the C7 vertebra. On the other hand, the larger transcripts may also be involved in the regulation of the *Hoxa5* proximal promoter by a mechanism that remains to be defined and their disruption by the *Hoxa6* mutation may affect the expression of the 1.8 kb transcript, that in turn impacts on skeletal patterning in the cervico-thoracic region.

## Discussion

### Multiple transcriptional units are present at the *Hoxa5* locus

This study aimed at characterizing the different transcriptional units encompassing the *Hoxa5* locus. Presence of multiple transcripts is not unique to the *Hoxa5* gene. Other *Hox* genes have been reported to produce several RNAs and in few cases, the molecular nature of these transcripts has been analyzed [4], [5], [47], [48]. In the case of the *Hoxa5*, *Hoxd4* and *Hoxb3* genes, the additional transcripts are expressed according to a *Hox*-like pattern but they have distinct boundaries of expression. Their generation involves multiple promoters, alternative splicing and/or the use of different polyadenylation sites. We have identified three promoters for the *Hoxa5* transcripts. The proximal one corresponds to the genuine *Hoxa5* promoter driving expression of the major 1.8 kb transcript. The larger transcripts are generated from either the distal D1 promoter, which is in fact the putative *Hoxa6* promoter, or the D2 promoter located downstream the 3′ extremity of the *Hoxa7* gene. Alternative splicing is also an important process contributing to the diversity of *Hoxa5* transcripts. Furthermore, the *Hoxa5* intron is not rigorously spliced in a small proportion of all *Hoxa5* transcripts adding to the number of RNA species detected. In contrast, the *Hoxa6* intron is systematically spliced in all transcripts that include the *Hoxa6* locus with the exception of the 11.0 kb form, which retains the intron sequences. Finally, only one polyadenylation site located at the end of the *Hoxa5* locus is utilized by all transcripts encompassing the *Hoxa5* and *Hoxa6* sequences. This data reveals the lack of a functional polyadenylation site at the *Hoxa6* locus. Thus in the e12.5 mouse embryo, the *Hoxa6* transcript exists only as a bicistronic gene product that can potentially generate two HOX proteins, HOXA5 and HOXA6.

Several studies have revealed the high transcriptional activity occurring along the mammalian *Hox* clusters [19]–[22]. Many of these transcriptional units are antisense to *Hox* genes. They are highly conserved between mouse and human, and some are polycistronic. For the *HoxA* cluster, a search with the UCSC genome browser indicates the existence of numerous sense and antisense transcripts covering the mouse *Hoxa5*-*Hoxa7* genomic region. Except for the *Hoxa5* 1.8 kb transcript, none of the larger transcripts characterized in the present work have been reported in databanks. However, portions of some of the transcripts listed include sequences of the *Hoxa5* larger forms. For instance, the first exon of the GenBank mRNA AK051552 corresponds exactly to the 5kb-*Hoxa5* transcript first exon. Twelve kb downstream in the *Hoxa4*-*Hoxa5* intergenic region, the second exon of the AK051552 transcript encompasses sequences corresponding to a distal *Hoxa3* exon annotated as the *Hoxa3* Y11717 mRNA (GenBank). The latter also initiates within the first exon of the 5 kb-*Hoxa5* transcript. In fact, the portrait of the different RNA species initiating in the *Hoxa6*-*Hoxa7* intergenic region reveals extensive transcription and production of large transcripts starting in the D2 promoter region and extending towards the vicinity of the *Hoxa3* gene. These data combined to ours argue for an intricate transcriptional activity at the D2 promoter. Interestingly, the integration of the Mouse Moloney Leukemia Virus (MMLV) nearby the D2 promoter in the *Hoxa6-Hoxa7* intergenic sequence impacts dramatically on the expression of the *Hoxa3* to *Hoxa10* genes [49]. Even though we cannot rule out the possibility of a long-distance perturbing effect from the MMLV enhancer on the *HoxA* promoters, it is tempting to speculate that the MMLV insertion predominantly affects the D2 promoter activity revealing the important role of the latter in the control of a subset of *HoxA* genes.

### Production of the HOXA5 and HOXA6 proteins

In *in vitro* assays, all the cDNAs corresponding to the larger *Hoxa5* RNA species tested can produce the 270 a.a. HOXA5 protein but not the 381 a.a. isoform initiating at the distal ATG (Fig. 1). However in HEK293 cultured cells, only the 1.8 kb transcript can be translated into the genuine HOXA5 protein. Moreover, expression of the HOXA5 protein along the mouse embryonic axis was not detected caudally of pv10, the anterior boundary of the expression domain of the larger transcripts. The concordance between the *Hoxa5* mutant phenotype and the expression domains of the HOXA5 protein and the 1.8 kb transcript support the notion that the 1.8 kb RNA is the functional *Hoxa5* transcript.

In the case of the HOXA6 protein, the bicistronic 5 kb-*Hoxa6*/a5 and 9.5 transcripts generate both HOXA5 and HOXA6 proteins *in vitro* but only the 5 kb-*Hoxa6*/a5 cDNA produces the HOXA6 protein in cultured cells. Whether the 5 kb-*Hoxa6/a5* transcript can generate the HOXA6 protein in the embryo remains to be defined via the development of a specific antibody. The absence of HOXA5 protein from the bicistronic 5 kb-*Hoxa6*/a5 unit is in accordance with the previous observations that polycistronic translation is a rare phenomenon in eukaryotes. In the case of polycistronic transcripts, the 5′ proximal cistron is usually the translated one as observed here [50]. Polycistronic transcription is not unusual in *Hox* clusters [19]. For instance, the *Hoxc4*, -*c5* and -*c6* genes are transcribed from a common promoter producing a primary transcript alternatively spliced to produce mature messengers encoding different proteins [51]. Finally, the presence of long 5′ untranslated region (UTR) sequences in the 5 kb-*Hoxa5* and 9.5 kb transcripts can explain the absence of translation as it can greatly reduce translational efficiency [50]. Thus, the molecular characterization of the different *Hoxa5* RNA species has unveiled an unexpected transcriptional organization and the promiscuity between the *Hoxa5* and *Hoxa6* loci in protein production.

### Role of the *Hoxa6* locus

Our studies raise questions about the role of the *Hoxa6* gene during development. Interestingly in teleosts, the *Hoxa6* gene is not present, most likely lost during the duplication process [52]. In mice, the *Hoxa6* mutation results in a mild skeletal phenotype resembling that of the *Hoxa5* mutants and occurring at an axial level located outside the *Hoxa6* expression domain. Indeed, the *Hoxa6* phenotype can be attributed to transcriptional interference that hinders transcription from the *Hoxa5* proximal promoter. In fact, the *neo* cassette used to mutate the *Hoxa6* gene was inserted in a temporal regulatory sequence responsible for the correct onset of *Hoxa5* expression supporting the notion that the presence of exogenous sequences nearby control regions impact on their efficiency [32], [44]. We cannot rule out the possible implication of the larger transcripts in the regulation of the *Hoxa5* proximal promoter and the effect their disruption by the *Hoxa6* mutation may have. To directly address this possibility would require the specific abolition of the larger transcripts in mice and the analysis of the phenotypic and molecular consequences. Taken together, our data suggest that it seems unlikely that the *Hoxa6* gene plays a role in axial specification, although it may serve other functions yet to be defined.

### Functional role of the *Hoxa5* 1.8 kb transcript

Along the antero-posterior axis, the HOXA5 protein is detected in the most-rostral subdomain of expression of the *Hoxa5* gene, which corresponds to the exclusive axial expression domain of the 1.8 kb transcript. In *Hoxa5* mutant mice, most of the defects observed lie within the HOXA5 protein expression domain. This further supports the importance of the 1.8 kb transcript as the biological effector of the *Hoxa5* gene during development.

Restricted HOX proteins expression was also reported for the *Hoxb4* and *Hoxb5* genes, and in both cases, the proteins were similarly localized in the anterior part of the gene expression domain [8], [15]. Comparatively to the RNA distribution, vertebrate HOX proteins may be more confined to precise axial levels, a situation comparable to what is observed for homeotic proteins in Drosophila embryos. Lots of efforts have been put on the regulatory mechanisms establishing the anterior boundary of *Hox* expression domains in vertebrates. Our findings enlighten the relevance of examining in more details how posterior boundaries may be fixed along the embryonic axes as well.

### Implication of the *Hoxa5* long noncoding RNAs

In e12.5 mouse embryo, the different polyA^+^ transcripts covering the *Hoxa5* coding sequences reported in this study originate from the DNA coding strand. Moreover, the larger transcripts have similar *Hox*-like expression profiles. They are expressed later during embryogenesis and in more posterior structures than the 1.8 kb transcript. Similarities between the expression profile of the larger transcripts and that of the *Hoxa7* gene indicate that they may share regulatory elements [38]. These long and interspersed transcripts also imply that DNA regions involved in *Hoxa5* gene regulation may be distributed along the cluster and emphasize the importance of the *Hox* cluster organization for the correct expression of *Hox* genes.

The larger *Hoxa5* transcripts cannot generate the HOXA5 protein. However, the 5 kb-*Hoxa6/a5* transcript can produce the HOXA6 protein in HEK293 cells. Thus, the 5kb-*Hoxa5*, the 9.5 and the 11.0 kb transcripts, all transcribed from the distal D2 promoter, can be considered as long noncoding RNAs. Both *Hoxa5* and *Hoxa6* null mutations disrupt the 5.0, 9.5 and 11.0 kb transcripts without any phenotypic consequence in the domain where they are expressed. These two mutations do not preclude the transcription of the transcripts but produce mutant versions 1 kb larger due to the presence of a *neo* cassette in each mutated locus. Thus, disruption of the larger transcripts does not impact on axial specification. Transcription of intergenic regions or upstream promoter sequences can affect the expression of adjacent genes, either by producing transcriptional interference, promoter competition for a limiting factor or by altering chromatin structure, leading to the hypothesis that the act of transcription *per se* of long noncoding RNAs is responsible for the regulatory effect [53]. Alternatively, long noncoding RNAs may regulate in *trans* gene expression as shown for HOTAIR, which participates to the Polycomb Repressive Complex 2 [20]. Our results in conjunction with the increasing sum of data on the potential role of long noncoding RNAs in transcriptional regulation now raise the following questions: do the *Hoxa5* larger transcripts represent “transcriptional noise” or do they contribute by themselves to the control of *Hox* gene expression [54].

New genome-wide technologies have unveiled the complex architecture of the eukaryotic transcriptome. The extensive overlap between transcriptional units, the existence of non-co-linear transcripts and the multifunctional roles of genomic sequences have even led to a re-evaluation of the current concept of the nature of the gene [55]–[57]. In this context and due to the fact that several of these features occur in *Hox* clusters, the latter appear as a paradigm from which we may learn more about the link existing between transcriptional complexity, functionality and genome organization.

## Materials and Methods

### Mouse strains and genotyping

The establishment of the *Hoxa5* mutant mouse line in the MF1-129/SvEv-C57BL/6 mixed background and the genotype by Southern analysis has been previously reported [23]. The *Hoxa6* mouse line in the 129/SvEv-C57BL/6 genetic background was provided by Dr. Mario Capecchi and genotyped by Southern blot analysis as described [44].

*Hoxa5* mutant mice were intercrossed with *Hoxa6* mutant mice to produce transheterozygous animals (*Hoxa5* ^+/−^; *Hoxa6* ^−/+^). For genetic background homogeneity, transheterozygous animals were interbred to generate mice carrying the possible *Hoxa5*; *Hoxa6* allelic combinations for subsequent skeletal analyses.

Embryonic age was estimated by considering the morning of the day of the vaginal plug as e0.5. All experiments were performed according to the guidelines of the Canadian Council on Animal Care and approved by the institutional animal care committee (Comité de Protection des Animaux du Centre Hospitalier Universitaire de Québec, CPA-CHUQ).

### PolyA^+^ RNA isolation and northern analysis

Total RNA from wild-type, *Hoxa5* ^−/−^ and *Hoxa6* ^−/−^ e12.5 embryos was isolated according to the TRIzol RNA extraction protocol (Invitrogen). For polyA^+^ RNA, the extraction was followed by two-step chromatography on oligo(dT)-cellulose column [58]. Seven µg of each polyA^+^ RNA preparation were used for northern analysis. Several genomic fragments covering the *HoxA* locus from *Hoxa7* to *Hoxa5* were used for antisense riboprobe synthesis (Fig. 2A). Hybridization to a GAPDH probe for quality control and quantitation was performed in parallel.

### 5′-Rapid Amplification of cDNA ends (RACE), 3′-RACE and RT-PCR analyses

The 5′ RACE protocol was essentially based on that described in [59]. The first-strand cDNA synthesis was performed with one µg of total RNA from e12.5 wild-type mouse embryos annealed to antisense *Hox* primer 1 (5′-GCGACCCTGCTATTGCCCAGACA-3′), primer 2 (5′-CTTCCGGTCGGTGCCTTCCTCAT-3′) or primer 3 (5′-CTGCGGGAGAAGCAGGCTGGAAT-5′; Fig. S1A). A polyA tail was added to the cDNAs. The dA-tailed cDNAs were first amplified using the nested *Hox* primer 1′ (5′-AACACAGCAGCCCCTGCACGGAA-3′), primer 2′ (5′-CTGCACGCTGCCGTCAGGTTTGT-3′) or primer 3′ (5′-GGCACCAGGGGGCAAAGCCAATA-3′) with an anchor primer complementary to the added oligo(dA) tail (5′-CCAGTGAGCAGAGTGACGAGGACTCGAGCTCAAGCTTTTTTTTTTTTTTTTT-3′) and an adapter primer included into the anchor primer (5′-CCAGTGAGCAGAGTGACGAGGAC-3′). A second round of amplification was set up with the primary PCR products and a different set of nested primers: primer 1″ (5′-CCCTCTTCCAGGGCTCAGGAA-3′), primer 2″ (5′-AAATGCGGCCGCCTGCTGCTCGGGAGAAAAGTG-3′) or primer 3″ (5′-AAATGCGGCCGCGGTCCCTGCACTGGGTCTAC-3′) with the anchoring primer (5′-GACGAGGACTCGAGCTCAAGC-3′). The secondary PCR products were subcloned prior to sequencing.

The 3′ RACE System for Rapid Amplification of cDNA Ends (GIBCO BRL) was used to identify the 3′ extremity of the *Hoxa5* transcripts. One µg of polyA^+^ RNA from e12.5 wild-type mouse embryos was used for the first-strand cDNA synthesis with a polyT adapter primer (5′-CCATCGATGTCGACTCGAGTTTTTTTTTTTTTTTTTT-3′) according to the protocol provided by the manufacturer. PCR amplification was then performed using a *Hoxa5* specific primer (5′-CTCCCCTTGTGTTCCTTCTG-3′) and a nested adapter primer (5′-CCATCGATGTCGACTCGAGT-3′) on an aliquot of the first-strand synthesis reaction. The resulting amplified products were subcloned and sequenced.

For the RT-PCR reactions, one µg of total RNA from e12.5 wild-type mouse embryos was used for the first-strand cDNA synthesis after annealing to an oligo(dT) primer or to the specific *Hox* primers 2 and 3 (described above). The following PCR reactions were then performed using various combinations of sense and antisense primers that all contain a *Not*I site. The sense primers are: primer 4 (5′-ATATGCGGCCGCCTCCGCTCCATCCTGCGTGCTT-3′), primer 5 (5′-AAATGCGGCCGCATCACAGTCCTGCAGAGGGGC-3′), and primer 6 (5′-AAATGCGGCCGCCACAAACGACCGCGAGCCACA-3′). The antisense primers are: primer 7 (5′-AAATGCGGCCGCCCCGGCGAGGATACAGAGGAT-3′) and primer 8 (5′-AAATGCGGCCGCACAGAGAGCTGCCCGGCTACT-3′). The RT-PCR products were then digested with *Not*I, subcloned and sequenced.

### Construction of *Hox/lacZ* transgenes and production of transgenic embryos

Genomic fragments encompassing the putative D1 and D2 promoter regions were subcloned in front of the IRES-βgeo cassette obtained from the pSA-IRESβgeolox2PGKDTA plasmid designed by Drs. Philippe Soriano and Valera Vasioukhin. We used a 4.19 kb *Eco*RI fragment extending from positions −7.96 kb to −3.77 kb (position +1 corresponding to the transcription start site of *Hoxa5* exon 1) for the D1 promoter and a 3.88 kb *Bgl*II-*Nru*I fragment extending from positions −11.46 kb to −7.58 kb for the D2 promoter. The 2.1 kb mesodermal enhancer (MES) was inserted upstream the promoter region in both constructs [32]. The constructs were made in pBluescript SKII+ (Stratagene) and purified following a cesium chloride centrifugation.

The Hox/lacZ sequences were isolated using a SalI-NotI digestion to remove vector sequences and they were purified on agarose gel. They were injected into the pronuclei of fertilized eggs derived from (C57BL/6 x CBA) F1 hybrid intercrosses following standard procedures [60]. Transgenic founder embryos were recovered from foster mothers at e12.5, genotyped by Southern analysis of yolk sac DNA using a lacZ specific probe to verify the integrity of the microinjected construct, and analyzed for lacZ expression by β-galactosidase staining as previously described [32].

### RNA *in situ* hybridization and immunofluorescence analyses

The whole-mount *in situ* hybridization protocol was based on the one described in [61], while radioactive *in situ* hybridization of paraffin sections was performed according to the protocol in [62]. The following murine genomic sequences were used as templates for synthesizing either digoxigenin or [^35^S] UTP-labeled riboprobes: a 830 bp *Bgl*II-*Hind*III fragment containing the 3′-untranslated region of the second exon of the *Hoxa5* gene (probe a), a 606 bp *Bgl*II-*Xho*I fragment present in the intergenic region between *Hoxa5* and *Hoxa6* genes (probe b), a 675 bp *Hind*III-*Eco*RI sequence located just downstream the *Hoxa7* gene (probe c), and a 356 bp *Kpn*I fragment present in the *Hoxa6* intron (probe d). The *in situ* experiments were performed on at least three specimens of each genotype.

Immunofluorescence staining with the rabbit anti-HOXA5 antibody and counterstaining with 4′,6-diamidino-2-phenylindole (DAPI; Molecular Probes) was performed as described in [46].

### Expression vectors, transfection, western and RT-PCR analyses

The sequence of the 1.8 kb cDNA was subcloned into the pcDNA3 expression plasmid (Invitrogen). A DNA fragment, from the p3XFLAG-MYC-CMV-24 expression vector (Sigma) and containing a MYC tag followed by the polyadenylation sequence of the human growth hormone gene, was inserted just before the stop codon of the HOXA5 protein using an overlapping PCR strategy with synthetic oligonucleotide primers covering the appropriate sequences [63]. A 5′-extended version of the 1.8 kb cDNA expression vector including the distal ATG codon in-frame with the HOXA5 open reading frame (ORF) was also designed. It contains upstream genomic sequences up to the *Eco*RV site at position −555 bp. This plasmid was made with and without the MYC tag. We also produced a pcDNA3 expression vector carrying the HOXA5-MYC version for the 5 kb-*Hoxa5* cDNA as well as for the 5 kb-*Hoxa6*/*a5* and for the 9.5 kb cDNAs. For these last two plasmids, a FLAG tag, from the p3XFLAG-MYC-CMV-24 vector, was added just before the HOXA6 stop codon. The TnT7 Quick coupled transcription-translation system (Promega) was used to produce [^35^S] methionine-labeled proteins according to the manufacturer's protocol. The translation products were analyzed by electrophoresis on a 12% sodium dodecyl sulfate-polyacrylamide gel (SDS-PAGE) and revealed by autoradiography.

HEK293 cells were transiently transfected by the calcium phosphate method in 60 mm petri dishes with 10 µg/dish of the *Hox* expression vectors [64]. The pEGFP-C2 expression vector was used to assess transfection efficiency (10 µg/dish; Clontech) and a pMEK1-MYC-FLAG plasmid (10 µg/dish; provided by Dr. Jean Charron) was used as a positive control for the immunodetection of the MYC and FLAG tags. Transfection of each plasmid was done in duplicate. Chloroquine was added at a final concentration of 50 µM. Three hours after transfection, the cells were shocked for 30 seconds at 37 °C with 15% glycerol in HEPES-buffered saline. Forty-eight hours after transfection, protein extracts were obtained after cell lysis in 300 µl of ice-cold lysis buffer (20 mM Tris-HCl pH 8.0, 1% NP-40, 10 mM EGTA, 5 mM MgCl_2_, 20 mM glycerol 2-phosphate, 25 mM NaF, 1 mM Na_3_VO_4_ and a proteinase inhibitor cocktail (Complete Mini EDTA-free; Roche Diagnostics)). After 15 minutes on ice, the extracts were centrifuged at 15,000 g at 4°C. Protein content of the supernatant was quantified using a Lowry-based assay (DC Protein Assay, Bio-Rad), and 20 µg of total protein lysate was resolved on a denaturing 12% SDS-PAGE, electrotransferred onto nitrocellulose (PALL) and probed overnight at 4°C with either a MYC-tag rabbit monoclonal antibody at a dilution of 1/1,000 (Cell Signaling Technology) or an anti-FLAG mouse monoclonal antibody at a dilution of 1/10,000 (Sigma) according to manufacturer's instructions. Membranes were also incubated with a mouse monoclonal anti-GAPDH antibody at a dilution of 1/20,000 (Fitzgerald Industries International) for loading control. Membranes were then incubated with the appropriate secondary horseradish peroxidase-conjugated antibody (a donkey anti-rabbit IgG at a dilution of 1/100,000 or a donkey anti-mouse at a dilution of 1/80,000; Jackson ImmunoResearch Laboratories). Proteins were revealed by chemiluminescence using the Western Lighting Plus-ECL system (PerkinElmer) according to manufacturer's protocol.

Total RNA from transfected HEK293 cells was isolated according to the TRIzol RNA extraction protocol (Invitrogen) and cDNA was synthesized with Superscript II Reverse Transcriptase (Invitrogen) using random primers. Reverse-transcription-PCR (RT-PCR) of the *Hoxa5-myc* portion was used to validate the expression of each transfected cDNA. PCR was performed for 25 cycles with an annealing temperature of 60°C. A 305 bp fragment was amplified with the *Hoxa5* forward primer 5′-CCCAGATCTACCCCTGGATG-3′ and the MYC-tag reverse primer 5′-GATGAGTTTTTGTTCGGGGC-3′.

### Skeletal analysis

Whole-mount skeletons were prepared from *Hoxa5*; *Hoxa6* compound newborn pups with Alcian blue for staining the cartilage and Alizarin red for staining the bone, as described in [27]. Skeletons were observed, and left and right sides of each vertebra were scored independently for bilateral markers.

## Supporting Information

Figure S1 Molecular characterization of the *Hoxa5* alternate transcripts by 5′-RACE and RT-PCR. (A) The transcription initiation site of the larger transcripts was determined by 5′-RACE. Genomic organization of the *Hoxa5*, *Hoxa6* and *Hoxa7* genes along the cluster is shown. Black, grey and open boxes indicate homeobox, translated, and transcribed sequences, respectively. The primers used are indicated (arrows). With primer 1, we obtained several clones showing that the 9.5 and 11.0 kb transcripts initiate in the *Hoxa6-Hoxa7* intergenic region at position −8905 bp. With primers 2 and 3, we obtained 5′-RACE products that revealed the presence of a 4.6 kb intron in the 5.0 kb transcript. The initiation site of this transcript also coincides with that of the 9.5 and 11.0 kb transcripts. A second population of clones was obtained with a transcription start site at position −4409 bp, which corresponds to the putative first base of *Hoxa6* exon 1. (B) By using various primer combinations in RT-PCR experiments, we established the molecular structure of the different transcripts. We also demonstrated that the 5.0 kb band detected by northern analyses include minor splicing variants and two major RNA species, one initiating at −8905 bp and a second starting at −4409 bp. The latter corresponds to the putative *Hoxa6* transcript. This transcript uses the polyA site of the *Hoxa5* gene. This result correlates with the absence of a functional polyadenylation sequence at the *Hoxa6* locus as shown by northen analysis. We also showed that splicing of the *Hoxa5* intron is not always complete and a low percentage of the *Hoxa5* transcripts contained intron sequences. A, *Acc*I; B, *Bg*lII; Ba, *Bam*HI; H, *Hind*III; K, *Kpn*I; RI, *Eco*RI; S, *Sac*I; St, *Stu*I; Xh, *Xho*I.
(0.26 MB PPT)

Figure S2 *Hoxa5* expression in the developing hindgut. Sections of e9.5 (A-C) and e10.5 (D-F) mouse embryos, and e15.5 (G-I), e17.5 (J–L) and DO (birth; M–O) hindgut tissues were hybridized with either probe a (B, E, H, K, N) or probe b (C, F, I, L, O). Bright-field views are shown on the left panels. (A–B) At e9.5, probe a detects *Hoxa5* transcripts along the gut up to the caudal foregut (arrowhead). (C) Expression with probe b is restricted to a more posterior region. (D–I) From e10.5 to e15.5, both probes reveal signal in the mesenchyme of the hindgut, while probe a detects *Hoxa5* transcripts in the myenteric plexi of the midgut (white arrow). (J–K) At e17.5, signal with probe a is confined to myenteric plexi in the proximal part of the hindgut (white arrow) while it still displays a diffuse mesenchymal expression in the distal hindgut. (M–N) Plexi of the enteric nervous system remain positive for probe a after birth as shown for D0. (L, O) No expression is observed with probe b from e17.5 onwards. d, distal hindgut; hg, hindgut; mg, midgut; p, proximal hindgut; tb, tailbud. Scale bar, 100 µm.
(6.19 MB TIF)
