# Supplementary material for: Multiple Promoters and Alternative Splicing: Hoxa5 Transcriptional Complexity in the Mouse Embryo
Source: PLoS One. 2010 May 12;5(5):e10600. doi: 10.1371/journal.pone.0010600 (PMC2868907; doi:10.1371/journal.pone.0010600)
Supplement: Figure S1 — Molecular characterization of the Hoxa5 alternate transcripts by 5′-RACE and RT-PCR. (A) The transcription initiation site of the larger transcripts was determined by 5′-RACE. Genomic organization of the Hoxa5, Hoxa6 and Hoxa7 genes along the cluster is shown. Black, grey and open boxes indicate homeobox, translated, and transcribed sequences, respectively. The primers used are indicated (arrows). With primer 1, we obtained several clones showing that the 9.5 and 11.0 kb transcripts initiate in the Hoxa6-Hoxa7 intergenic region at position −8905 bp. With primers 2 and 3, we obtained 5′-RACE products that revealed the presence of a 4.6 kb intron in the 5.0 kb transcript. The initiation site of this transcript also coincides with that of the 9.5 and 11.0 kb transcripts. A second population of clones was obtained with a transcription start site at position −4409 bp, which corresponds to the putative first base of Hoxa6 exon 1. (B) By using various primer combinations in RT-PCR experiments, we established the molecular structure of the different transcripts. We also demonstrated that the 5.0 kb band detected by northern analyses include minor splicing variants and two major RNA species, one initiating at −8905 bp and a second starting at −4409 bp. The latter corresponds to the putative Hoxa6 transcript. This transcript uses the polyA site of the Hoxa5 gene. This result correlates with the absence of a functional polyadenylation sequence at the Hoxa6 locus as shown by northen analysis. We also showed that splicing of the Hoxa5 intron is not always complete and a low percentage of the Hoxa5 transcripts contained intron sequences. A, AccI; B, BglII; Ba, BamHI; H, HindIII; K, KpnI; RI, EcoRI; S, SacI; St, StuI; Xh, XhoI. (0.26 MB PPT) [file pone.0010600.s001.ppt]

## Slide 1
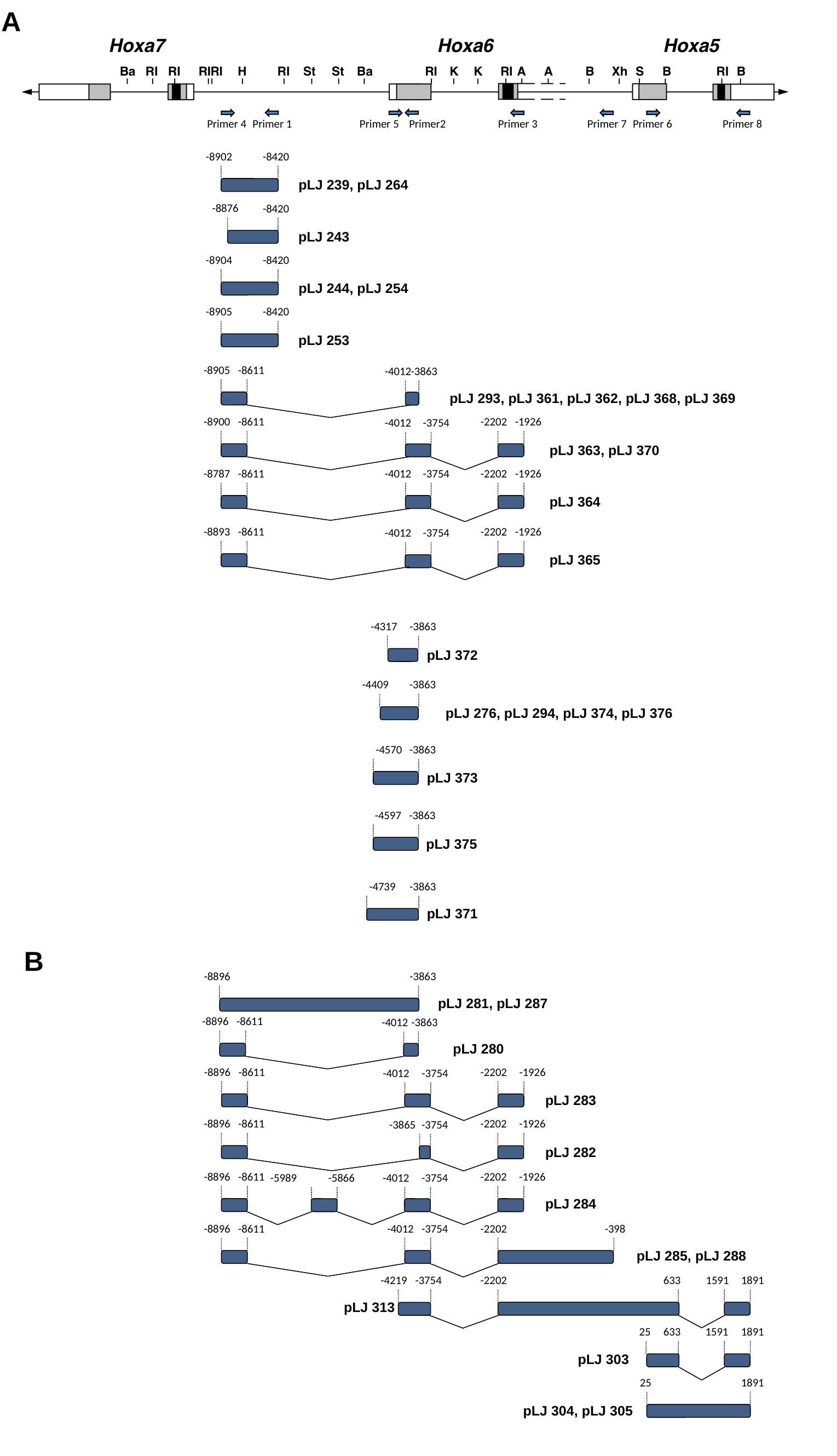

A
Primer 4
Primer 1
Primer 5
Primer2
Primer 3
Primer 7
Primer 6
Primer 8
-8902
-8420
pLJ 239, pLJ 264
-8876
-8420
pLJ 243
-8904
-8420
pLJ 244, pLJ 254
-8905
-8420
pLJ 253
-8905
-8611
-4012
-3863
pLJ 293, pLJ 361, pLJ 362, pLJ 368, pLJ 369
-8900
-8611
-2202
-1926
-4012
-3754
pLJ 363, pLJ 370
-8787
-8611
-4012
-3754
-2202
-1926
pLJ 364
-8893
-8611
-2202
-1926
-4012
-3754
pLJ 365
-4317
-3863
pLJ 372
-4409
-3863
pLJ 276, pLJ 294, pLJ 374, pLJ 376
-4570
-3863
pLJ 373
-4597
-3863
pLJ 375
-4739
-3863
pLJ 371
B
-8896
-3863
pLJ 281, pLJ 287
-8896
-8611
-4012
-3863
pLJ 280
-8896
-8611
-2202
-1926
-4012
-3754
pLJ 283
-8896
-8611
-2202
-1926
-3865
-3754
pLJ 282
-8896
-8611
-2202
-1926
-5989
-5866
-4012
-3754
pLJ 284
-8896
-8611
-4012
-3754
-2202
-398
pLJ 285, pLJ 288
1591
1891
-4219
-3754
-2202
633
pLJ 313
1591
1891
25
633
pLJ 303
1891
25
pLJ 304, pLJ 305
