# Supplementary figures and images for: Multiple Promoters and Alternative Splicing: Hoxa5 Transcriptional Complexity in the Mouse Embryo
Source: PLoS One. 2010 May 12;5(5):e10600. doi: 10.1371/journal.pone.0010600 (PMC2868907; doi:10.1371/journal.pone.0010600)

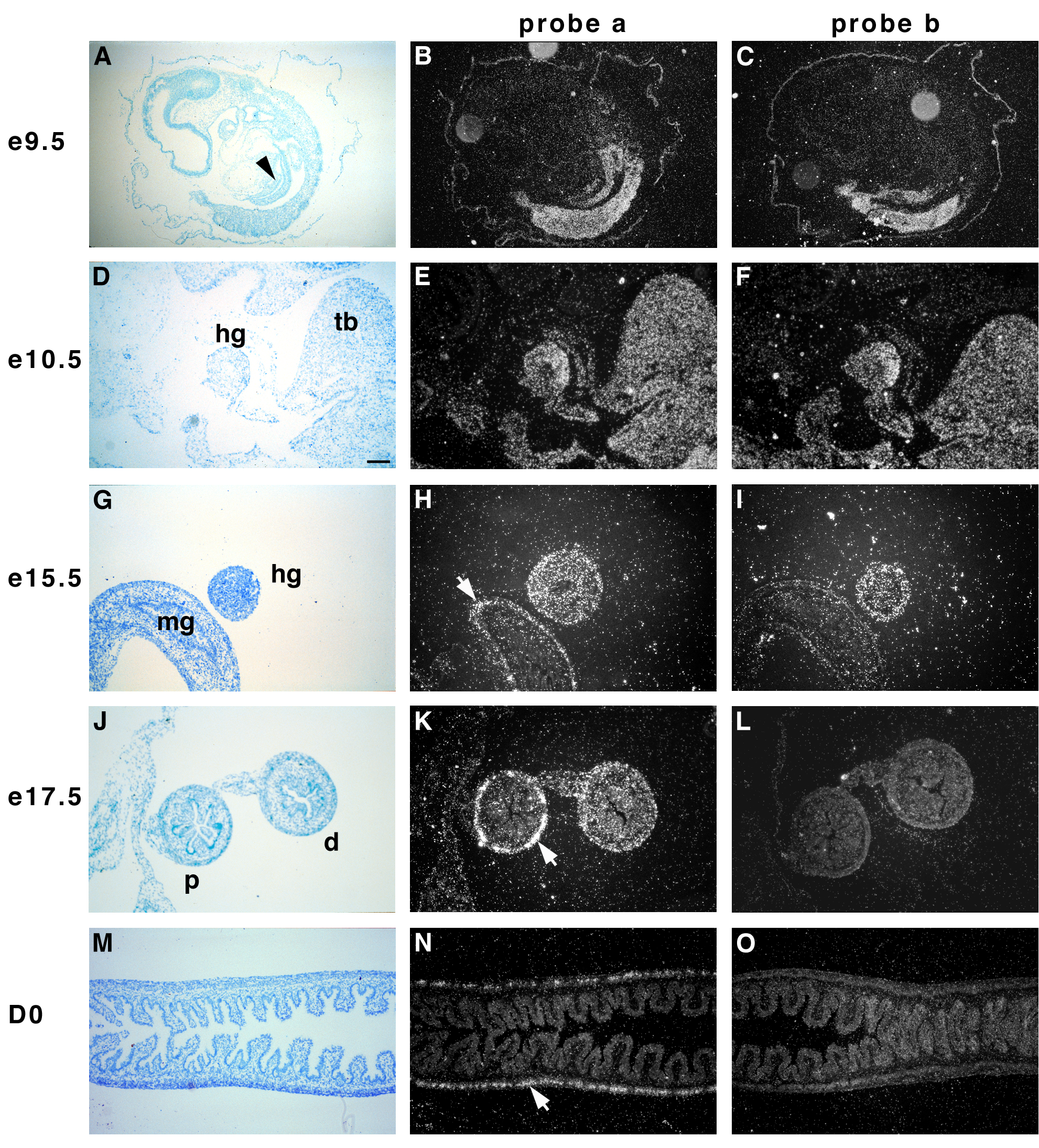

Supplement: Figure S2 — Hoxa5 expression in the developing hindgut. Sections of e9.5 (A-C) and e10.5 (D-F) mouse embryos, and e15.5 (G-I), e17.5 (J–L) and DO (birth; M–O) hindgut tissues were hybridized with either probe a (B, E, H, K, N) or probe b (C, F, I, L, O). Bright-field views are shown on the left panels. (A–B) At e9.5, probe a detects Hoxa5 transcripts along the gut up to the caudal foregut (arrowhead). (C) Expression with probe b is restricted to a more posterior region. (D–I) From e10.5 to e15.5, both probes reveal signal in the mesenchyme of the hindgut, while probe a detects Hoxa5 transcripts in the myenteric plexi of the midgut (white arrow). (J–K) At e17.5, signal with probe a is confined to myenteric plexi in the proximal part of the hindgut (white arrow) while it still displays a diffuse mesenchymal expression in the distal hindgut. (M–N) Plexi of the enteric nervous system remain positive for probe a after birth as shown for D0. (L, O) No expression is observed with probe b from e17.5 onwards. d, distal hindgut; hg, hindgut; mg, midgut; p, proximal hindgut; tb, tailbud. Scale bar, 100 µm. (6.19 MB TIF) [file pone.0010600.s002.tif]
